# Childhood Acute Respiratory Infections and Household Environment in an Eastern Indonesian Urban Setting

**DOI:** 10.3390/ijerph111212190

**Published:** 2014-11-25

**Authors:** Tomoyuki Shibata, James L. Wilson, Lindsey M. Watson, Alyse LeDuc, Can Meng, Ansariadi, Ruslan La Ane, Syamsuar Manyullei, Alimin Maidin

**Affiliations:** 1 Public Health Program, Northern Illinois University, DeKalb, IL 60115, USA; E-Mails: lwatson566@gmail.com (L.W.); alyse.leduc1@gmail.com (A.L.); Z1653061@students.niu.edu (C.M.); 2 Institute of the Study for Environment, Sustainability, and Energy, Northern Illinois University, DeKalb, IL 60115, USA; E-Mail: jwilson41@niu.edu (J.W.); 3 Faculty of Public Health, Universitas Hasanuddin, Makassar, South Sulawesi 90245, Indonesia; E-Mails: ansariadi@gmail.com (A.); ruslan_fkm@yahoo.com (R.L.A.); manongkoki@yahoo.com (S.M.); aliminmaidin@gmail.com (A.M.); 4 Department of Geography, Northern Illinois University, DeKalb, IL 60115, USA; 5 Division of Statistics, Northern Illinois University, DeKalb, IL 60115, USA

**Keywords:** childhood pneumonia, acute respiratory infections, household environment, particulate matter, low and middle income countries

## Abstract

This pilot study evaluated the potential effect of household environmental factors such as income, maternal characteristics, and indoor air pollution on children’s respiratory status in an Eastern Indonesian community. Household data were collected from cross-sectional (*n* = 461 participants) and preliminary childhood case-control surveys (pneumonia cases = 31 diagnosed within three months at a local health clinic; controls = 30). Particulate matter (PM_2.5_ and PM_10_) was measured in living rooms, kitchens, children’s bedrooms, and outside areas in close proximity once during the case-control household interviews (55 homes) and once per hour from 6 a.m. to midnight in 11 homes. The household survey showed that children were 1.98 times (*p* = 0.02) more likely to have coughing symptoms indicating respiratory infection, if mothers were not the primary caregivers. More children exhibited coughing if they were not exclusively breastfed (OR = 2.18; *p* = 0.06) or there was a possibility that their mothers were exposed to environmental tobacco smoke during pregnancy (OR = 2.05; *p* = 0.08). This study suggests that household incomes and mother’s education have an indirect effect on childhood pneumonia and respiratory illness. The concentrations of PM_2.5_ and PM_10_ ranged from 0.5 to 35.7 µg/m^3^ and 7.7 to 575.7 µg/m^3^, respectively, based on grab samples. PM was significantly different between the case and control groups (*p* < 0.01). The study also suggests that ambient air may dilute indoor pollution, but also introduces pollution into the home from the community environment. Effective intervention programs need to be developed that consider multiple direct and indirect risk factors to protect children.

## 1. Introduction

Globally, the leading cause of death in children under the age of five is pneumonia, an acute respiratory infection [1,2]. Every year, more than one million young children die from pneumonia, a figure that is larger than the total number of child deaths attributed to AIDS, malaria, measles, meningitis, injuries and all other post-neonatal conditions combined [3]. Low and middle-income countries in South-East Asia and sub-Saharan Africa are responsible for the more than 70% of pneumonia-associated child deaths in the world [1]. Vaccination has been the primary public health intervention for pneumonia. However, vaccination is only effective against *Streptococcus pneumonia* (or pneumococcal) and* Haemophilus influenza type b* (Hib), which are two common microbial etiologic agents for pneumonia. As a result, pneumonia cannot be prevented by vaccination if it is caused by respiratory syncytial virus or other pathogens [1,2]. Poor access to vaccinations for the public, especially for low income households, is also another major concern.

Alternatively, public health interventions may focus on reducing modifiable risk factors associated with childhood pneumonia. Childhood pneumonia has been considered a disease of poverty [4], which is difficult to overcome. Recent studies using meta-analysis show that several definite risk factors are related to either the host (e.g., malnutrition, low birth weight (≤2500 g), and lack of measles immunization within the first 12 months) or household environment (e.g., maternal characteristics, crowding, and indoor air pollution) [1,4]. Globally, approximately one-half of premature deaths are attributable to pneumonia associated with particulate matter (PM), which is a major air pollutant [5]. Recent studies conducted in a low-income country (Bangladesh) demonstrated increased risk of acute lower respiration infections as a result of indoor PM exposure [6,7]. Common sources of indoor PM include tobacco smoking and burning biomass (e.g., wood) for cooking. PM exposure has also been recognized as a contributor to increased mortality and morbidity from cardiovascular illnesses and lung cancer [3,8,9,10,11,12].

Indonesia is a lower-middle-income and the third most populous country (approximately 250 million) in Asia, which covers South and East Asia [13]. Although the largest leading cause of death in Indonesian children under the age of five include prematurity (20%) followed by acute respiratory infections (17%) [14], to date there has been little investigation focusing on risk factors associated with childhood acute respiratory infections. It is important to investigate risk factors in Indonesia in order to develop, improve, and promote a site-specific environmental public health intervention. In this exploratory study, previously defined environmental risk factors associated with acute respiratory infections were examined in an Indonesian urban setting. Although incomes and maternal characteristics are considered to be socioeconomic factors, this study defined them as factors within the social environment [4].

The study’s null hypotheses are that there would be no statistically significant differences in income, maternal characteristics, and indoor air quality between cross-sectional survey households with children, regardless of acute respiratory infection (and related symptoms) status, as well as no statistically significant differences between case and control households. Another null hypothesis is that concentrations of indoor PM concentration would not vary significantly over time.

## 2. Materials and Methods

This study took place in Makassar (population of 1.3 million), South Sulawesi, which is the largest city in eastern Indonesia. No previous studies have been conducted regarding environmental household factors associated with children’s acute respiratory infections. The study used two different types of study designs reflected in their respective data collection methods: a cross-sectional study based on a household survey of the general population and a preliminary survey of a case-control group that included PM monitoring. The survey data were used to evaluate multiple household environmental risk factors including income levels, maternal characteristics, and indoor air quality. The City of Makassar and the Northern Illinois University Institutional Review Board (HS11-0170) approved the study prior to any data collection.

### 2.1. Cross-Sectional Surveys

Household information regarding income, maternal characteristics (e.g., caregiver, exclusive breastfeeding, education), sources of indoor air pollution (e.g., energy sources for cooking and parental smoking), and children’s respiratory status was obtained from the general population that included a wide socioeconomic range of households (*n* = 1184) using a convenience sampling approach conducted by face-to-face interviews in June 2011 [15]. The current study was restricted to only 461 households (38.9%), which had at least one child less than 12 years at the time of survey. All survey participants were at least 18 years old with an average (*x*) age of 34.8 years (sd ± 8.5). Almost all participants were married (96.7%) with 2.2% single and 1.1% divorced. The distribution of male and female participants was 60.9% and 39.1%, respectively. In order to determine the incidence of childhood pneumonia and respiratory symptoms (e.g., asthma attack, coughing, headache, and sneezing), the participating parents were asked if their children had been diagnosed with pneumonia and/or experienced any respiratory symptoms at any point during the previous two weeks prior to the interview [15]. If there was more than one child in the household, the participants provided answers about the youngest child’s health status.

### 2.2. Preliminary Case-Control Study

Government-operated community health centers, or “Pusat Kesehatan Masyarakat” (Puskesmas) identified households with children under the age of five, who had been diagnosed with pneumonia within the last three months, with 31 of them agreeing to participate in this study as a members of the case group. Puskesmas physicians, who typically do not have access to X-ray equipment, diagnose pneumonia based on symptoms that are consistent with the World Health Organization (WHO)’s diagnostic criteria for pneumonia: coughing and or difficult breathing with or without fever, and the presence of either fast breathing or lower chest wall in-drawing [3]. For the control group, 30 households with children who had not been diagnosed with pneumonia were recruited in the same neighborhood as the case-households. A total of 61 households (*i.e.*, 51.6% case and 48.4% control) were identified. The case-control household survey included income, maternal characteristics, and sources of indoor air pollution. This study was conducted in June 2012.

It should be noted here that this was a preliminary case-control study supported by a local puskesmas. The small sample size (e.g., minimum *n* = 50) was justified based on the alternative hypothesis that the incidence of childhood pneumonia would be significantly different between low income and relatively higher income households because it was considered to be a disease of poverty [4]. Since there were no records linking childhood illnesses to household income locally, the information needed for sample size calculation was obtained from a recent study conducted in the same city. This study demonstrated a strong association between lower household monthly incomes (less than IRD (Indonesian Rupiah) 1,520,000 or USD 152) per month and childhood diarrhea, another poverty associated disease [15].

### 2.3. Residential PM Measurement

Fine (PM_2.5_) and coarse (PM_10_) particles at three indoor locations (*i.e.*, living room, kitchen, child’s bedroom) and one outside of the house (e.g., patio or in the alley) was evaluated in June (dry season), 2012 using two 831 Four Channel Handheld Particle Counters (Met One Instruments, Grants Pass, OR, USA). Among 61 preliminary case-control survey households, 55 households (*n* = 27 cases and 28 controls) allowed the research team to perform a set of one-time measurements of PM during the interview. Single measurements collected in this manner were referred to as grab-samples to distinguish them from other sampling efforts. PM was also monitored once every hour from 6 a.m. to midnight in the same four places described above in the eight households (*i.e.*, seven cases and one control), in which PM was measured by grab sampling during the interview. This hourly sampling was conducted by adult household members. Three additional households with children (<5 years old) who had not been diagnosed with pneumonia participated in PM hourly sampling as control group.

### 2.4. Statistical Analysis

Microsoft Excel 2010 was used to record, store, and organize all survey results and PM measurement data. IBM SPSS Statistics 20 was used for descriptive analysis (e.g., mean and median), *t*-test (e.g., comparison of household incomes), Non-parametric test (e.g., comparison of education levels), Odds ratios, (e.g., measuring associations) and Pearson Chi-Square test (e.g., determination of the significance) or the Fisher exact test, when the expected cell size was less than five [16].

## 3. Results

### 3.1. Children’s Respiratory Status

The cross-sectional household survey (*n* = 461) showed that 0.4% of youngest children (average age 2.4 ± 1.4 years old) had pneumonia in the past two weeks prior to the interview. The equivalent incidence rate of pneumonia was 113 cases per 1000 people per year, which was comparable to the incidence rates of childhood respiratory illness (e.g., 57 in Antara and 671 in Tamalanrea sub-districts) reported in Makassar [17]. Many children had coughs (15.4%), headaches (9.1%) and sneezing (7.4%) in the past two weeks. Since pneumonia cases were low, coughing was used as an indicator for acute respiratory infection [3] in the cross-sectional survey.

The preliminary case-control household survey (*n* = 61) included children who presented typical symptoms of acute respiratory infections such as coughing (54.1%), sneezing (52.5%), and headache (16.4%) during the two weeks prior to the survey. Case households (with children under five diagnosed with pneumonia) comprised 51.6% of the household survey participants. Those children diagnosed with pneumonia in the previous months were 14.4 times (95% CI 4.1–50.3; *p* < 0.01) more likely to still have coughing symptoms and 8.03 times more likely (95% CI 2.55–25.3; *p* < 0.01) to have sneezing symptoms in subsequent months. Subsequent headaches in the following months were not associated with a previous pneumonia diagnosis (*p* = 0.18).

### 3.2. Household Income

The median household income was approximately IDR 2,000,000 per month (or USD 200/month) as self-reported from the cross-sectional and case-control surveys (Table 1). Five percent of the households in Makassar had a monthly income less than IDR 600,000, which is one of several criteria that define poverty in Indonesian social statistics [18]. Much larger proportions of households in the city were lower than IDR 1,520,000, a low-income household criteria based on the international standard of USD 1.25 per day for a household with an average size of four family members in Makassar [15]. Within the cross-sectional household survey, there was no significant difference in income levels between those households that had children with coughing symptoms and those that did not (*p* = 0.47–0.78). There was no significant difference in incomes between households with puskesmas diagnosed pneumonia in children or without (*p* = 0.50–0.92).

### 3.3. Maternal Characteristics

Primary caregivers for young children were commonly mothers (cross-sectional household survey: 81.5%; case-control household survey: 70.5%). However, the cross-sectional household survey showed that children were 1.98 times (95% CI 1.09–3.59; *p* = 0.02) more likely to have coughing, if their mothers were not the primary caregivers. Such an association was not observed in the case-control survey (*p* = 0.52).

**Table 1:** Household environment from the cross-sectional and preliminary case-control surveys.

Household Environment	Cross-Sectional		Preliminary Case-Control	
	Overall *n* = 459	*p*-value (Coughing)	Overall *n* = 61	*p*-value (Pneumonia)
Household income				
Median, IDR	1,950,000	0.62	2,000,000	0.50
<IRD 1,520,000	209 (45.3) *	0.47	19 (31.3)	0.92
<IRD 600,000	24 (5.2)	0.78	3 (4.9)	0.61
Mother is primary caregiver	368 (80.1)	0.02	43 (70.5)	0.52
Exclusive breast feeding to an infant	82 (18.6)	0.06	--	--
Mother’s education				
Less than elementary school	15 (8.3)	0.37	2 (3.4)	1.00
Less than junior high school	46 (25.6)	0.25	9 (15.5)	1.00
Energy for cooking				
Electricity	55 (11.9)	0.56	1 (1.6)	1.00
Propane	418 (90.7)	<0.01	57 (93.4)	1.00
Kerosene	59 (12.8)	0.23	10 (16.4)	0.51
Wood	19 (4.1)	0.49	4 (6.6)	0.61
Someone smokes inside house	299 (70.9)	0.44	36 (60.0)	0.11
Parental smoking				
Mother	4 (2.8)	1.00	3 (5.5)	0.61
Father	177 (70.5)	0.79	2 (66.7)	1.00
Smoking				
Nearby a woman	100 (56.5)	0.14	--	--
Nearby a pregnant woman	55 (31.1)	0.08	--	--
Nearby a child	73 (41.5)	0.56	--	--

Notes: Cross-sectional and preliminary case-control surveys used different methods for different purposes. Thus, results from these two surveys were not statistically compared. In the table, * The number within a parenthesis represent percentage (%) of participants in the category. The p-values for the cross-sectional and preliminary case-control survey from *t*-test and chi-square analysis for households with and without respiratory symptoms in children. -- indicates no data.

The World Health Organization (WHO) recommends exclusive breastfeeding for six months [19]. Only 18.4% of the households in the cross-sectional household survey exclusively breastfed their children. Children had an elevated likelihood of coughing (OR = 2.18; 95% CI 0.96–4.97; *p* = 0.06), if they were not exclusively breastfed while infants.

The average education level for female participants, who were assumed to be mostly mothers of children, was a high school degree. One fourth of mothers did not complete the nine years of compulsory education (through junior high school) and some did not possess an elementary education. Education levels for mothers were not significantly different between households with and without children coughing (*p* = 0.25) or pneumonia (*p* = 1.00).

### 3.4. Source of Indoor Air Pollution

Multiple energy sources were used in the cross-sectional survey households. More than 90% of these households used propane, while there were a few households that used kerosene and wood, which produces more toxic fumes than other energy types (e.g., electricity and gas) used for cooking. The use of unhealthy cooking energy sources did not show any significant associations for children with coughing symptoms (*p* = 0.23 and 0.49 for kerosene and wood, respectively) and pneumonia (*p* = 0.51and 0.61 for kerosene and wood, respectively).

A majority of adult males (70.5%) smoked cigarettes, while only a small percentage of women (2.8%) smoked in the cross-sectional population. Many smokers did not mind smoking cigarettes nearby women (56.5%), pregnant women (31.1%), and children (41.5%). More children exhibited coughing (OR = 2.05; 95% CI 0.91–4.63; *p* = 0.08) if their mothers presumably had been exposed to secondhand smoke or environmental tobacco smoke (ETS) during pregnancy. All other smoking-related data showed no statistical association with a child coughing (*p* = 0.14–1.00).

### 3.5. Residential PM Exposure

The concentrations of indoor PM were not statistically different from locations just outside of the house or ambient PM measurements among the 55 preliminary case-control households measured by grab-sampling (*p* = 0.23 and 0.57 for PM_2.5_ and PM_10_, respectively) and the 11 households sampled hourly (*p* = 0.71 and 0.51 for PM_2.5_ and PM_10_, respectively) (Table 2). PM levels at the four sampling household locations were also not statistically different from each other (*p* = 1.00). In eight houses, in which PM was measured by both grab (GS) and hourly sampling (HS), the levels of PM_2.5_ were significantly different from each other (*p* = 0.04), while PM_10_ levels were not statistically different (*p* = 0.51). Although this study did not measure PM over a long-term period, some residential areas had relatively high levels of PM that may exceed the WHO Guidelines for PM_2.5_ (*i.e.*, annual mean = 10 μg/m^3^ and 24-h mean = 25 μg/m^3^) and PM_10_ (annual mean = 20 μg/m^3^ and 24-h mean = 50 μg/m^3^) [20].

**Table 2:** Indoor and ambient particulate matter (PM).

PM	Overall PM (µg/m^3^) Range with an Average	Indoor PM (µg/m^3^)	Ambient PM (µg/m^3^)	*p*-value
PM_2.5_ (GS)	0.5–35.7, *x* = 7.7 ± 4.9	7.9 ± 4.8 (*n* = 163)	7.0 ± 5.0 (*n* = 55)	0.23
(HS)	0.5–197, *x* = 10.4 ± 12.8	9.2 ± 10.1 (*n* = 538)	9.6 ± 19.3 (*n* = 135)	0.71
PM_10_ (GS)	7.7–576, *x* = 50.2 ± 45.1	49.2 ± 26.9 (*n* = 163)	53.2 ± 77.5 (*n* = 55)	0.57
(HS)	1.2–618, *x* = 48.8 ± 53.2	44.1 ± 53.8 (*n* = 538)	40.7 ± 46.6 (*n* = 135)	0.51

Note: GS and HS stand for grab and hourly sampling methods.

Hourly sampling showed significant differences in PM concentrations between households with current and previous cases of children diagnosed with pneumonia (*x* = PM_2.5_ 11.8 ± 14.8 µg/m^3^ and PM_10_ 55.5 ± 62.2 µg/m^3^; *p* < 0.01 for both) and control households (PM_2.5_ 7.8 ± 6.7 µg/m^3^ and PM_10_36.0 ± 24.1 µg/m^3^; *p* < 0.01 for both) (Table 3). On the other hand, GS PM levels were not significantly different between case and control groups (*p* = 0.94 for PM_2.5_ and 0.21 for PM_10_).

**Table 3:** Particulate matter (PM) in households with childhood pneumonia (case) and control.

PM	Average PM (µg/m^3^)		
	Case	Control	*p*-value
PM_2.5_ (GS)	7.7 ± 4.6	7.7 ± 5.1	0.94
(HS)	11.8 ± 14.8	7.8 ± 6.7	<0.01
PM_10_ (GS)	46.3 ± 54.9	54.0 ± 32.7	0.21
(HS)	55.5 ± 62.2	36.0 ± 24.1	<0.01

Note: GS and HS stand for grab and hourly sampling methods.

**Figure 1:**
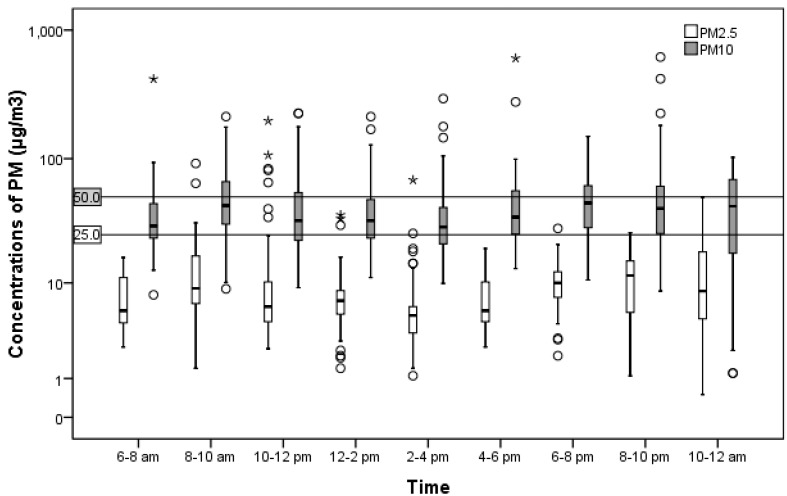
Change in particulate matter (PM) concentrations in a residential setting.

Note: Particulate matter 25 and 50 μg/m^3 ^are the WHO Guidelines for PM_2.5_ and PM_10_ 24-h means, respectively (WHO, 2006).

Two peaks were observed between 8 to 10 a.m. and 6 to 8 p.m. These peaks coincided with rush hours as well as cooking breakfast and dinner (Figure 1). Temporal associations were not determined in this study.

## 4. Discussion

The present study is a preliminary effort to explore and evaluate the potential association between a child’s respiratory status and his or her household environment including income, maternal characteristics, and indoor air pollution in an urban setting in eastern Indonesia. An initial cross-sectional study is employed to uncover the more prevalent and salient and factors involved in local childhood respiratory health status. This study was then followed up with a pilot case-control study using several factors from both the local survey and the literature in addition to measuring household PM.

### 4.1. Household Income

Childhood pneumonia has been considered a disease of poverty that contributes to high child mortality and morbidity in low- and middle-income countries [4]. Similarly, in urban settings of an upper-middle income country like Brazil, significantly higher cases of pneumonia are experienced in children living in low income areas when compared to children in neighboring high income areas [21]. Household income for the cross-sectional study population did not show a significant association with the incidence of acute respiratory infections in children. The results did show that children are still at risk of acute respiratory infection regardless of household income level. Although the sample size for this preliminary case-control study was initially thought to be large enough (power = 0.99) to detect a strong association between household income and a poverty-related childhood disease, the actual sample size was too small (power = 0.06). This was due to the fact that p0: the proportion of exposure among controls was almost the same as p1: the proportion of exposure among cases.

The cross-sectional study focused on the general population of Makassar, excluding the lowest income population living in slums. Low household incomes were assumed to be linked to crowding (e.g., living in slums and or larger number of people living in a household), which is a risk factor for childhood pneumonia [1]. Diarrhea, which is a concomitant disease with childhood pneumonia, is also a likely risk factor [1]. Children in Makassar have been reported to be 3.21 times more likely to develop diarrhea if households had income levels below $152/month (95% CI 1.40–7.35; *p* < 0.01) [11]. Therefore, this study still considered low households income as an indirect factor for childhood acute respiratory infections rather than as a direct cause or factor. In the future, a full-scale study needs to focus on smaller statistical differences between childhood pneumonia and risk factors that would be based on a larger sample size.

### 4.2. Maternal Characteristics

Mothers play a critical role in childhood acute respiratory infections [4]. They are typically the primary caregivers, but older female siblings often take on this role while their mothers are at work. Since older girls may lack the experience of their mothers, the results show that children had an increased risk of coughing, if their mothers were not primary caregivers. This finding was consistent with Rudan *et al.*’s contention that caregiver experience level is a likely factor in childhood pneumonia [1].

Mother’s education did not show a significant association with childhood acute respiratory infection in the results, which is inconsistent with earlier studies [4,22]. On the other hand, the mother’s educational status can affect their perception of child health and is related to improper hygienic behaviors [4,15]. Watson *et al.* found that young children are 4.72 times (95% CI 1.22–18.3; *p* = 0.03) more likely to develop diarrhea if mothers washed their hands without soap after defecation [15]. The lack of a primary education also has a negative influence a mother’s hygienic behaviors and results in an increased risk of a concomitant disease like pneumonia in their children [11]. Thus, the study considers mother’s education as an additional, but indirect factor for childhood respiratory infections.

The present study’s results show that mothers not breastfeeding exclusively may increase the risk of childhood acute respiratory infections. This finding supports Rudan [1] *et al.* claim that non-exclusive breastfeeding is a definite risk factor for pneumonia [1]. Although exclusive breastfeeding for infants has been promoted by the local and provincial health departments (Drs. Harymin, Apt. M. Kes) personal communication, 9 June 2014), the study raises a concern for the lack of its practice in Makassar.

### 4.3. Sources of Indoor Air Pollution

Burning cooking fuels is a major source of indoor air pollution in low- and middle-income countries. Approximately one-half of the world’s population uses some form of biomass for cooking. Biomass sources have been implicated in adverse health effects for children (e.g., pneumonia and other acute respiratory infections) in low- and middle-income countries [23,24,25,26,27,28,29]. Kerosene is more commonly used than wood in the present study site, but exposure to this source may damage lung function and increase the risk of infectious diseases [30]. The non-significant association between children’s respiratory status and energy types evaluated in this study was consistent with an earlier study in the Czech Republic, which is considered a high income country [31]. In the current study, 78.1% of the households opened the windows while cooking, but there was no significant difference in children’s respiratory symptoms between households with or without opened windows (*p* = 0.30). Opened windows are quite common in Indonesia due to its tropical climate where toxic fumes from burning wood or kerosene may be diluted by natural ventilation. However, it should be noted here that the PM measurements were conducted during the early dry season in Sulawesi.

Tobacco smoking is another major source of indoor air pollution. Approximately 80% of tobacco smokers live in low and middle income countries and Indonesia is no exception [32]. Environmental tobacco smoking (ETS) and parental smoking is considered to be a likely factor for respiratory illnesses in children [2,31,33,34,35]. It should be remembered that approximately 70% of adult males in Makassar smoke cigarettes and they don’t mind smoking near children and women, including pregnant women. It could be hypothesized that young children spend more time with mothers than fathers, while pregnant women spend substantial time with their husbands. Thus, the presence of smokers did not show a statistical association with children’s respiratory status, while an elevated risk of coughing was observed in children, if their mothers had been exposed to ETS during their pregnancy. This result was consistent with an earlier study demonstrating that prenatal exposure affects children’s lower respiratory health [31].

### 4.4. Residential PM Exposure

PM is a major component of indoor air pollution [29]. Recent studies conducted in both a high-income (*i.e.*, the U.S.) and a low-income country (*i.e.*, Bangladesh) showed that increases of indoor PM affects children’s respiratory symptoms [6,30]). A recent cohort-study conducted in Bangladesh showed that exposure to indoor PM_2.5_ higher than 100 µg/m^3^ would increase risk of acute lower respiratory infections among infants [6]. Although the average concentration for indoor PM_2.5_ in this study was approximately 10 µg/m^3^, households with children recently diagnosed with respiratory symptoms had higher PM_2.5_ and PM_10_ levels than the control group. While earlier studies showed that densities for indoor and outdoor PM were different [8,9], the present study demonstrated no significant differences between indoor and outdoor densities. PM measurements support the previous hypothesis that opening windows dilutes pollution from indoor sources, but ambient air pollution from the surrounding community could impact indoor air quality simultaneously. Accordingly, the study suggests that ambient air could play a major role in childhood respiratory illness in low-middle income countries, especially in tropical environments.

Certain limitations emerged in the conduct of the present study due to resource constraints, including time. The instruments used in this study were not capable of measuring PM continuously, failing to include the complete range of possible indoor PM levels throughout the day. Measurements were conducted during the dry season, when air exchange between indoor settings and outside may be less impeded. Thus, the complete range of daily and seasonal air quality measurements, especially which for PM_2.5_, is not known for this study site. A future study of residential PM exposure is recommended that uses multiple sampling or continuous monitoring methods instead of grab-sampling. A more comprehensive, long term study of air quality and children’s respiratory health would also include the examination of geographic correspondence between PM density and childhood respiratory acute respiratory infections using spatial analytical tools.

Specific sources of PM, such as ETS, burning cooking fuels, and traffic will continue to be important in furthering understanding of PM exposures. Currently, ambient air quality monitoring is carried out twice a year in Makassar, the largest city in eastern Indonesia (Zulkifli, S.T. Amran, personal communication, 10 June 2014). This study represents a preliminary effort in the evaluation of the effects of PM on childhood respiratory health and the promotion of increased monitoring efforts.

## 5. Conclusions

Within an eastern Indonesian urban context, this pilot study demonstrates that childhood acute respiratory infections are potentially the result of various direct risk factors, such as caregiver’s experience, breastfeeding, maternal ETS exposure, and household PM level, which are similar to factors found in other low and medium-income countries. Although some of the null hypotheses posited here could not be rejected due to methodological limitations, the study still suggests that some risk factors like household income and mother’s education have an indirect effect on childhood pneumonia. It will be important to conduct a larger follow up study using a case-control or cohort study design with continuous measurements of indoor PM in order to capture the full range of air quality measurements and their association with childhood respiratory health outcomes over time and space. Such an effort will improve scientific basis for developing specific locally tailored environmental public health intervention programs in Indonesia with the potential to be extended to other low- and middle income countries.
